# Comparison of percutaneous transforaminal endoscopic decompression and short-segment fusion in the treatment of elderly degenerative lumbar scoliosis with spinal stenosis

**DOI:** 10.1186/s12891-021-04804-6

**Published:** 2021-10-28

**Authors:** Pengfei Li, Yuexin Tong, Ying Chen, Zhezhe Zhang, Youxin Song

**Affiliations:** Department of Orthopedic, Affiliated Hospital of Chengde Medical University, No. 36 Nanyingzi St, Chengde, 067000 Hebei China

**Keywords:** Degenerative lumbar scoliosis, Spinal stenosis, Percutaneous transforaminal endoscopic decompression, Fusion, Geriatric patients

## Abstract

**Background:**

Degenerative lumbar scoliosis (DLS) combined with spinal stenosis is increasingly being diagnosed in the elderly. However, the appropriate surgical approach remains somewhat controversial. The aim of this study was to compare the results of percutaneous transforaminal endoscopic decompression (PTED) and short-segment fusion for the treatment of mild degenerative lumbar scoliosis combined with spinal stenosis in older adults over 60 years of age.

**Methods:**

Of the 54 consecutive patients included, 30 were treated with PTED and 24 were treated with short-segment open fusion. All patients were followed up for at least 12 months (12–24 months). Patient demographics, and perioperative and clinical outcomes were recorded. Visual analog scale (VAS) scores, Oswestry disability index (ODI) scores, and modified Macnab criteria were used to assess clinical outcomes. At the same time, changes in disc height, segmental lordosis, coronal Cobb angle, and lumbar lordosis were compared.

**Results:**

The mean age was 68.7 ± 6.5 years in the PTED group and 66.6 ± 5.1 years in the short-segment fusion group. At 1 year postoperatively, both groups showed significant improvement in VAS and ODI scores compared with preoperative scores (*p* < 0.05), with no statistically significant difference between groups. However, VAS-Back and ODI were lower in the PTED group at 1 week postoperatively (*p* < 0.05). According to the modified Macnab criteria, the excellent rates were 90.0 and 91.6% in the PTED and short-segment fusion groups, respectively. However, the PTED group had a significantly shorter operative time, blood loss, postoperative hospital stay, postoperative bed rest, and complication rate. There was no significant difference in radiological parameters between the two groups preoperatively. At the last follow-up, there were significant differences in disc height, segmental lordosis at the L4–5 and L5–S1 levels, and Cobb angle between the two groups.

**Conclusion:**

Both PTED and short-segment fusion for mild degenerative lumbar scoliosis combined with spinal stenosis have shown good clinical results. PTED under local anesthesia may be an effective supplement to conventional fusion surgery in elderly patients with DLS combined with spinal stenosis.

## Introduction

Degenerative lumbar scoliosis (DLS) is a deformity of the spine that occurs after skeletal maturation. It is defined as a Cobb angle >10° in the coronal plane with degenerative changes of the intervertebral discs and facet joints [1]. DLS most often occurs in old age [2]. Its prevalence shows an increasing trend as the population ages [3]. Spinal stenosis is defined as any type of narrowing of the spinal canal, nerve root canal, or intervertebral foramina [4]. DLS, as part of the body’s aging process, is often accompanied by spinal stenosis [5]. Additionally, coronal deformities can cause stenosis on the concave side of the lumbar spine [5, 6], which in turn brings about low back pain, radicular pain and intermittent claudication [7]. This phenomenon complicates nerve compression and makes surgical treatment difficult.

DLS combined with spinal stenosis can greatly affect the quality of life of patients [8, 9]. Nonsurgical approaches, such as physical therapy, NSAIDs, and steroid injections, have proven difficult to achieve the desired results [10]. At this point, surgery may be the treatment of choice for patients with DLS. However, the surgical treatment strategy for scoliosis combined with stenosis remains controversial: decompression alone, short-segment fusion, or long-segment fusion [11–13]. The treatment decision becomes even more important in elderly patients with combined spinal deformity [10]. This is because it is associated with a higher complication rate and mortality in elderly patients [14]. At the same time, we must not only consider the stiffness of the lumbar spine of elderly patients with DLS, which makes it difficult to achieve the best correction effect. It should also be noted that osteoporosis in elderly patients weakens the strength of internal fixation, which can easily lead to loss of correction and pseudoarthrosis [7]. Several previous studies [11, 15, 16] have reported that long-segment fusion may not be necessary in patients with degenerative scoliosis with a Cobb angle of 10°-30°. Considering this characteristic of the elderly, simple decompression and short-segment fusion without orthopedic goals may be the appropriate treatment.

PTED has the advantages of low anesthetic risk, minimal trauma, and rapid recovery. In recent years, it has achieved promising results in the treatment of elderly patients with degenerative lumbar spondylolisthesis with spinal stenosis [17, 18]. However, it has rarely been reported in the treatment of DLS combined with spinal stenosis. For decompression alone, a minimally invasive PTED procedure for the treatment of elderly patients with Cobb angle 10°-30° DLS combined with LSS may be a good attempt. The purpose of this study was to observe the results of the PTED procedure and short segment fusion for the treatment of mild DLS combined with spinal stenosis in elderly individuals and to provide a reference for clinical practice.

## Methods and materials

### Participants

A total of 54 patients were retrospectively included between June 2017 and June 2020. All patients provided written consent. Our hospital institutional review board approved the study. In this study, short-segment fusion refers to one-level or two-level fusion. All surgeries were performed by the same surgical team. The inclusion criteria were as follows: (1) imaging diagnosis of DLS combined with spinal stenosis; (2) Cobb angle in the coronal plane of 10°-30°; (3) age greater than or equal to 60 years; (4) presentation of unilateral nerve root symptoms; and (5) failure of conservative treatment for more than 3 months. The exclusion criteria were as follows: (1) main symptom of low back pain; (2) preoperative dynamic radiographs showing significant segmental instability; (3) history of previous lumbar spine surgery; and (4) pathological conditions such as tumor, trauma, and infection. Preoperative demographic characteristics, perioperative conditions, and clinical outcomes were recorded.

### Surgical procedure

Clarify the segment of responsibility: The responsible segment is identified preoperatively by symptoms, signs and imaging findings. However, for patients for whom it is difficult to make a definitive diagnosis, we typically use a diagnostic nerve root block. One milliliter of 2% lidocaine is injected around the suspected nerve under the guidance of the C-arm. If the lower extremity pain is fully or partially relieved, then that segment is considered the responsible segment.

Short-segment fusion group: General anesthesia is selected, and a posterior median incision is performed after disinfecting the towel. The structure of the responsible segment is fully revealed, and attention is given to protecting the supraspinous ligament and joint capsule of the nonfixed segment. The pedicle screw is inserted with the aid of C-arm X-ray machine fluoroscopy. Based on the preoperative imaging data, the articular eminence and lamina of the responsible segment are selectively resected and adequately decompressed. The discs of the responsible segment are then removed, and intervertebral and posterior posterolateral bone grafting is performed. Bilateral titanium rods are installed. Moderate bracing or compression is applied to restore local alignment. Finally, the pedicle nail is tightened.

PTED group: Local infiltration anesthesia is selected, and the surgical position is lateral. Orthotropic fluoroscopy requires adjustment of the bilateral pedicles to a symmetrical position based on the responsible segment, vertebral rotation, and lateral subluxation. Lateral fluoroscopy should avoid double shadowing. A working channel is created with the assistance of a C-arm X-ray machine and connected to a percutaneous transforaminal endoscopic spine system (Maxmore spine, Germany). Under endoscopy, the hypertrophic ligamentum flavum, ventral part of the hypertrophic upper articular process, and extruded intervertebral disc tissue is removed. Finally, osteophytes on the posterior edge of the vertebral body are selectively removed. Endoscopy shows that the nerve root is obviously pulsating with the heartbeat, and the operation ends after hemostasis is sufficient.

### Measures

Evaluation of imaging parameters: Before and after the operation, the patient's disc height, segmental lordosis, coronal Cobb angle, and lumbar lordosis angle are measured.

Evaluation of clinical results: VAS and ODI are used to evaluate clinical results before the operation, and 1 week, 3 months, and 12 months after the operation. In addition, a modified Macnab criterion is used to assess surgical satisfaction at the final follow-up.

**Fig. 1:**
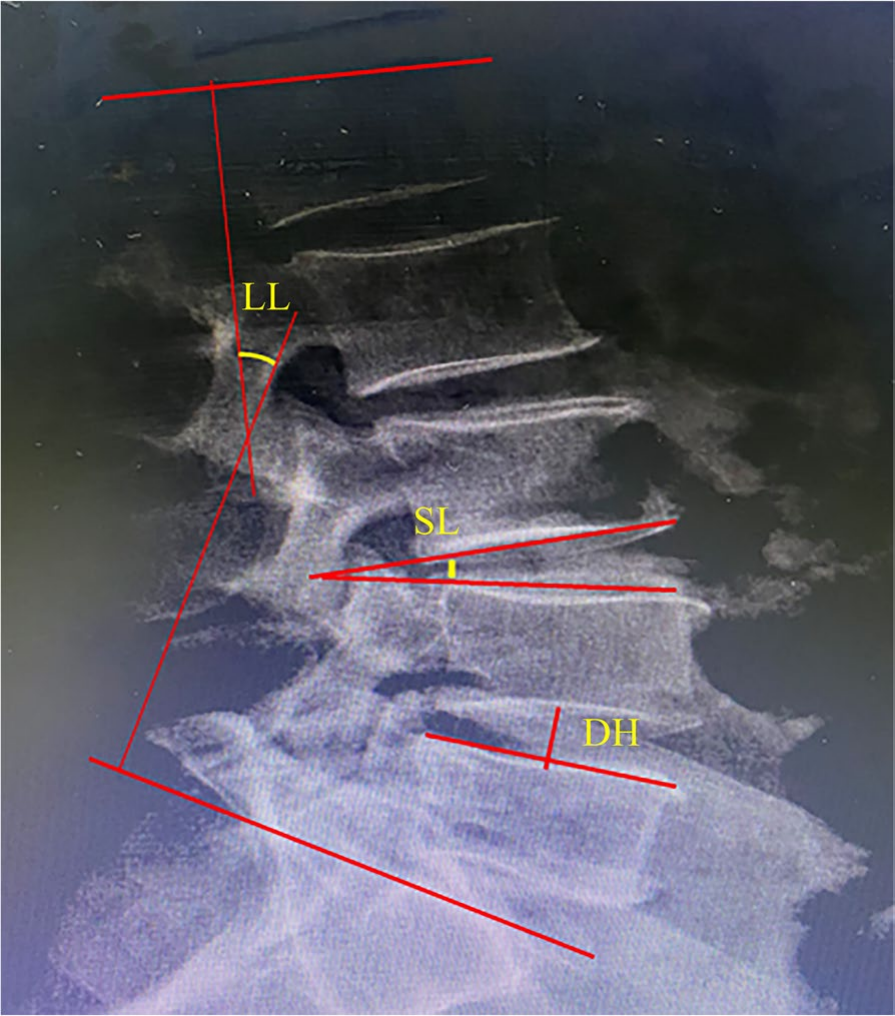
DH: disc height; SL: segmental lordosis; LL: lumbar lordosis

Definition of radiological parameters (Fig. 1): 1) Disc height: The distance between the two points that are the intersections of the superior and inferior vertebral endplates and the bisector of the inferior endplate line segment; 2) Segmental lordosis: angle between the inferior end plate of the vertebral body above and superior end plate of the vertebral body below; 3) Lumbar lordosis: angle between the superior endplate line of L1 and the superior endplate line of S1.

### Statistical assessments

Statistical calculations were performed using the SPSS 26 program (IBM, Armonk, USA). Demographic and radiological values and clinical outcomes of the two groups of patients were analyzed using the chi-square test, Student's t test, and Mann–Whitney U test. The significance level was defined as *p* < 0.05.

## Results

### Preoperative demographic characteristics and outcomes

The average follow-up time was at least 12 months (12–24 months). A total of 54 patients participated in this study. In the PTED group, there were 8 males and 22 females, with an average age of 68.7±6.5 years. There were 7 males and 17 females in the short-segment fusion group, with an average age of 66.6±5.1 years. The comorbidities of the two groups were similar, the most common being hypertension. There was no significant difference in the level or number of levels operated on in the fusion and PTED groups. These demographic characteristics are summarized in Table 1. In addition, there were no significant differences in disc height, segmental lordosis, Cobb angle, or lumbar lordosis between the two groups before surgery (Table 2).

**Table 1:** Demographic characteristics of the short-segment fusion group and PTED group

Items	Short-segment fusion	PTED	***p*** Value
Number of patients	24	30	
Age (years)	66.6±5.1	68.7±6.5	0.287
Male/female	7/17	8/22	
Levels involved			0.405
L3–4	10(27.0%)	7(15.9%)	
L4–5	20(54.1%)	25(56.8%)	
L5–S1	7(18.9%)	12(27.3%)	
Number of levels (n/%)			0.584
Single level	11(45.8%)	16(53.3%)	
Two levels	13(54.2%)	14(46.7%)	
Comorbidities (n/%)			
Cardiovascular	14(58.3%)	17(56.7%)	
Cerebrovascular	4(16.7%)	5(16.7%)	
Endocrinologic	4(16.7%)	6(20.0%)	
Pulmonary	3(12.5%)	3(10.0%)	
Bones and Joints	5(20.8%)	7(23.3%)	
Others	3(12.5%)	4(13.3%)

**Table 2:** Changes in radiographic parameters in the short-segment fusion group and PTED group

Items	Short-segment fusion		PTED	
	Preoperative	Final follow-up	Preoperative	Final follow-up
Disc height (mm)				
L3–4	8.0±1.4	9.8±0.9^*^	7.9±1.5	7.8±1.4^**!**^
L4–5	8.3±1.5	10.2±1.1^*^	8.1±1.6	8.0±1.5^**!**^
L5–S1	8.2±1.4	9.7±1.2^*^	8.2±1.5	8.2±1.4^**!**^
Segmental lordosis (°)				
L3–4	4.3±2.4	4.4±2.3	4.4±2.5	4.4±2.4
L4–5	6.4±3.1	9.4±2.7^*^	6.5±2.8	6.4±2.6^**!**^
L5–S1	13.1±3.6	15.6±3.9^*^	13.1±3.1	12.5±2.5^**!**^
Cobb angle (°)	13.9±3.1	8.3±3.8^*^	15.8±4.7	16.4±5.0^**!**^
Lumbar lordosis (°)	38.6±12.4	40.7±11.8	36.1±13.0	36.4±12.4

* Significantly different from preoperative parameters (*P* < 0.05)

! Significantly different from the short-segment fusion group (*P* < 0.05)

### Clinical results

As shown in Table 3, the PTED group had a significantly shorter operative time (211.3±14.2 vs. 76.8±16.3), less blood loss (414.2±113.6 vs. 13.2±3.8), a shorter postoperative hospital stay (13.3±2.5 vs. 6.5±3.2) and shorter postoperative bed rest (3.4±0.8 vs. 0.5±0.2) than the short-segment fusion group (*P* < 0.05). Three cases of incisional infection, one case of transient sensory disturbance, one case of intraoperative dural tear, and one case of lower extremity venous thrombosis occurred in the short-segment fusion group. In the PTED group, one patient experienced recurrence and underwent a second fusion procedure at 2 months postoperatively, and two other patients experienced transient sensory disturbance.

**Table 3:** Operation characteristics of the two groups

Outcome measure	Short-segment fusion	PTED	***p*** Value
Operation time (min)	211.3±14.2	76.8±16.3	<0.05
Bleeding quantity (mL)	414.2±113.6	13.2±3.8	<0.05
Length of postoperative stay (d)	13.3±2.5	6.5±3.2	<0.05
Time to ambulation (d)	3.4±0.8	0.5±0.2	<0.05
Major complication			
Revision surgery	0	1	
Wound infection	3	0	
Transient dysesthesia	1	2	
Intraoperative dura tear	1	0	
Thrombus formation	1	0	

**Fig. 2:**
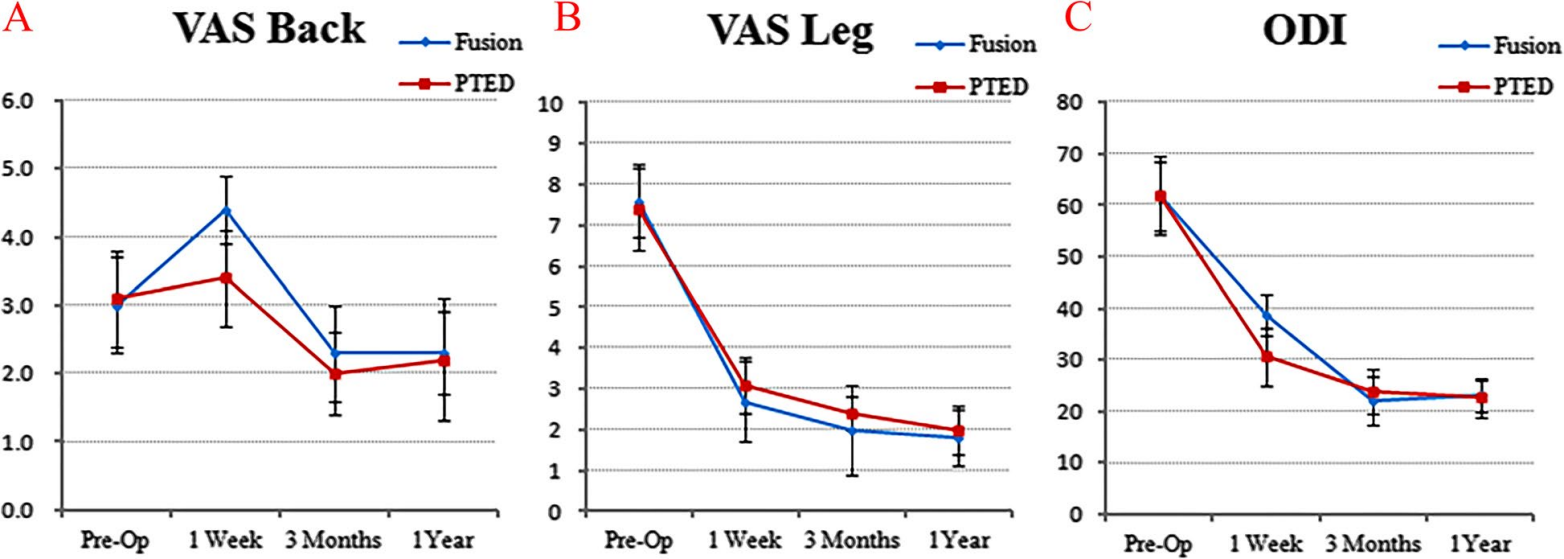
The clinical outcomes of the PTED group and the short-segment fusion group at different follow-up time points. **A** VAS back pain score. **B** VAS leg pain score. **C** Oswestry Disability Index

The VAS and ODI scores of the two groups are shown in Fig. 2. The mean preoperative VAS back pain score was not significantly different between the PTED and short-segment fusion groups, at 3.1 ± 0.7 and 3.0 ± 0.7, respectively. However, at 1 week postoperatively, it was 3.4 ± 0.7 and 4.4 ± 0.5, respectively, which was a statistically significant difference (*P* < 0.05). There was no significant difference at 3 months postoperatively or at 1 year postoperatively, and it was significantly better than preoperatively (*P* < 0.05). The average preoperative VAS leg pain scores of the PTED group and the short-segment fusion group were 7.4±0.9 and 7.6±1.0, respectively. They were reduced to 2.0±0.7 and 1.8±0.6 at 1 year postoperatively. There was no significant difference in the average preoperative ODI scores between the two groups, and they were significantly improved 1 year after surgery. However, at 1 week after surgery, the ODI scores of the PTED group and the short-segment fusion group were 30.6±5.5 and 38.8±4.1, respectively, with significant differences between the groups (*P* < 0.05).

**Fig. 3:**
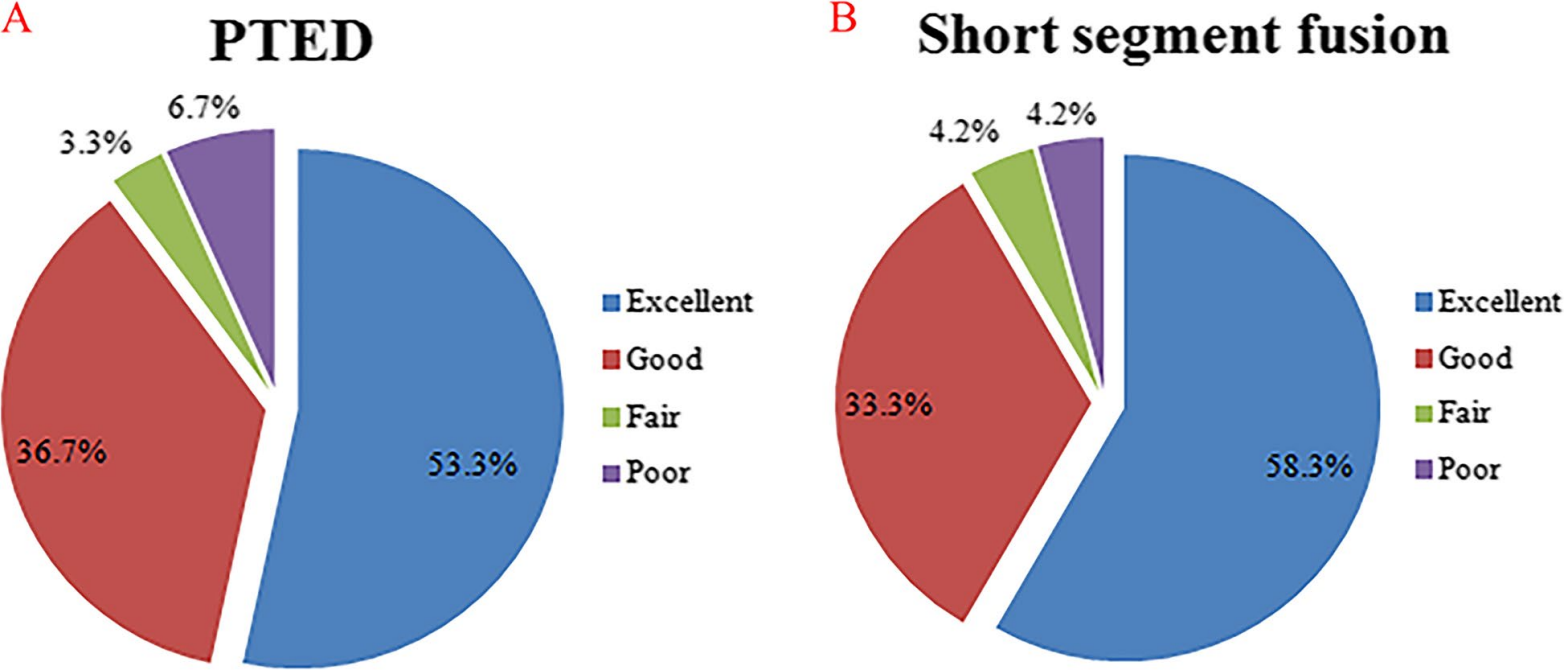
Clinical outcomes of the PTED group and the short-segment fusion group at the last follow-up

As shown in Fig. 3, according to the modified Macnab criteria, the excellent/good rates were 90.0 and 92.6% in the PTED and short-segment fusion groups, respectively.

**Fig. 4:**
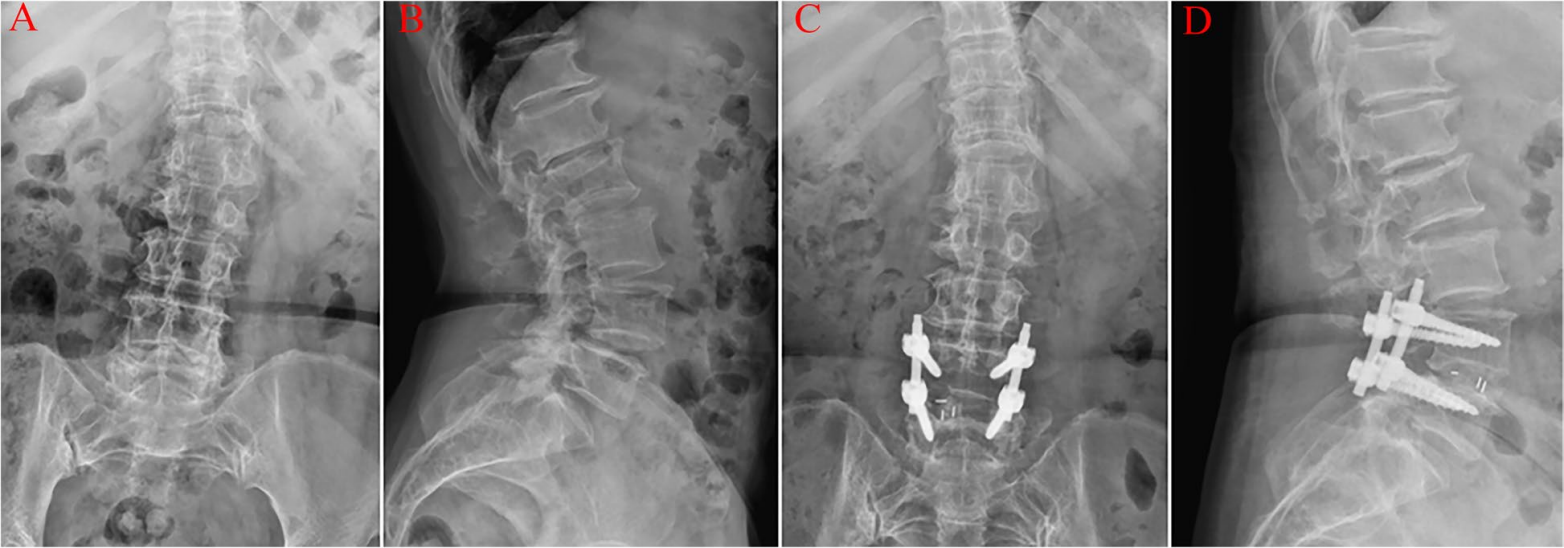
A 66-year-old female patient. **A** and **C** are the frontal X-rays of the lumbar spine before and after the operation, whereas **B** and **D** are the lateral X-rays of the lumbar spine before and after the operation

As shown in Fig. 4, the Cobb angle of the coronal plane in the short-segment fusion group was corrected to a certain extent, and the lumbar lordosis angle did not change significantly.

**Fig. 5:**
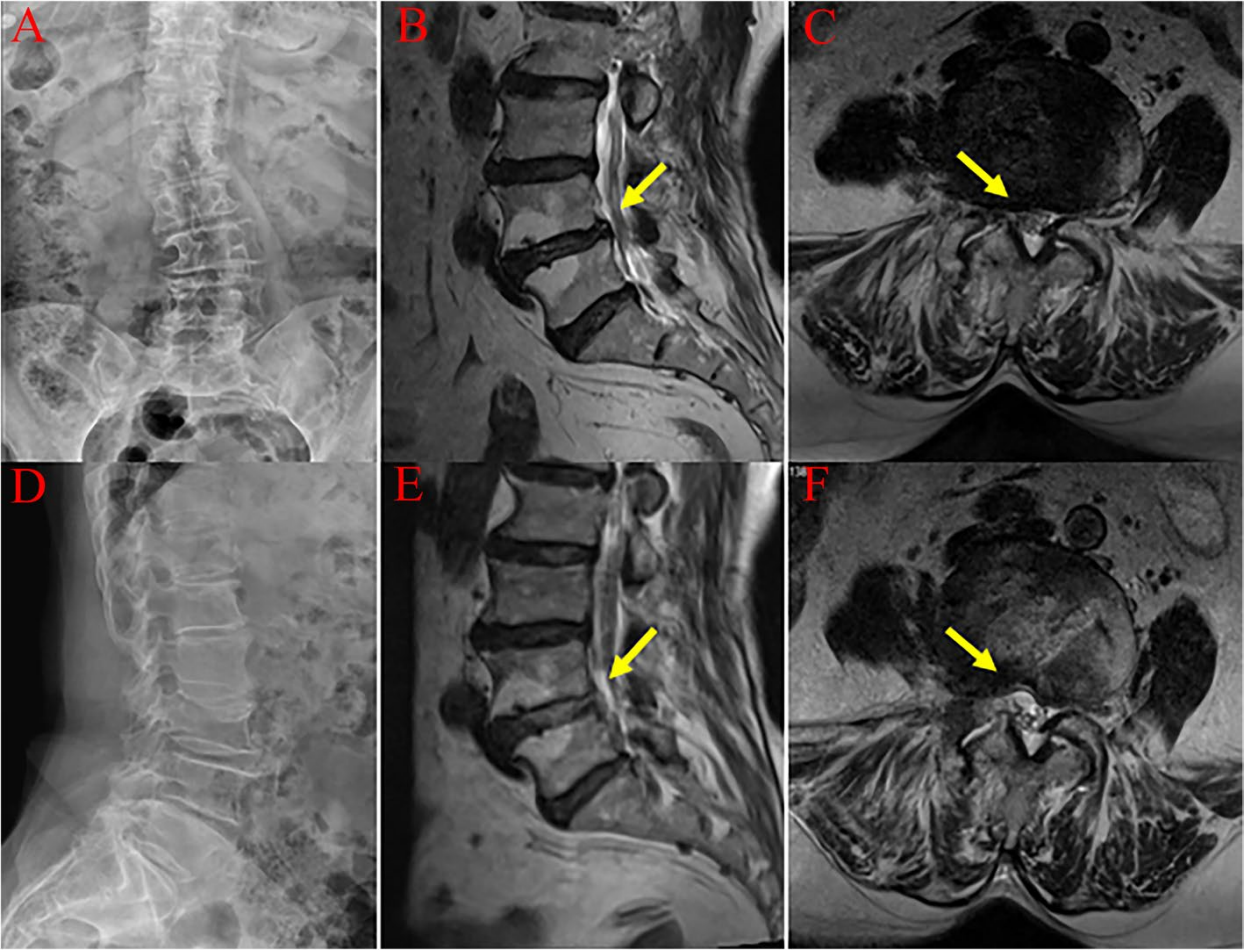
A 73-year-old male patient with a 1-year history of previous cerebral infarction. He had severe degeneration of the lumbar spine and underwent PTED surgery on the L4–5 segment. **A** and **D** are preoperative frontal and lateral radiographs, respectively. **B** and **E** are sagittal comparisons of MRI before and after surgery, respectively. **E** and **F** are axial comparisons of MRI images of the responsible segment. (Yellow arrows show the changes in the L4–5 segment before and after surgery)

As shown in Fig. 5, PTED surgery can be performed thorough nerve root decompression by removing the proliferative and cohesive facet joints, hypertrophic ligamentum flavum, extruded intervertebral disc tissue and some osteophytes.

### Radiological results

At the final follow-up, disc height, segmental lordosis at the L4–5 and L5–S1 levels, and Cobb angle were significantly improved in the short-segment fusion group compared with the preoperative period. No significant changes were observed in the PTED group before and after surgery. In addition, at the last follow-up, there were significant differences in disc height, segmental lordosis at the L4–5 and L5–S1 levels, and Cobb angle between the two groups.

## Discussion

DLS has become a common condition in the context of a continuing global aging population. McAviney et al [19] reported that the prevalence of degenerative scoliosis over 60 years old was 36%. Aging causes degenerative changes in the body's skeletal structure and intervertebral discs. Asymmetric disc degeneration leads to the development of DLS and loss of anterior lumbar lordosis [20, 21]. Not all patients with DLS have symptoms. However, elderly DLS patients with spinal stenosis often suffer from low back pain, sciatica, and intermittent claudication [2]. Surgery is the final choice for most patients with DLS who do not respond to conservative treatment. Compared with younger patients, elderly patients usually have severe spinal degeneration, worse surgical endurance, and a higher risk of complications [22]. Therefore, the best surgical treatment is still controversial.

Most scholars [11, 15, 16] have stated that decompression alone and short-segment fusion are sufficient for patients with degenerative scoliosis with a Cobb angle of 10°-30°. Usually, short-segment fusion is a common option for the treatment of single- or two-segment degenerative disease. It does not involve the correction and fusion of the entire scoliosis curve but rather the fixed fusion of a single decompressed region. This technique is an appropriate option for mild to moderate scoliosis and mild subluxation of the parietal spine. Simmons et al [15] indicated that long-segment fusion is not required to correct scoliosis deformities. When spinal balance is maintained by short fusion, symptoms of low back pain and stenosis may disappear. Lee et al [23] performed a study of short-segment fusion for mild DLS and found that clinical and radiographic outcomes remained satisfactory 5 years after surgery. Theoretically, the use of short-segment fusion for mild DLS combined with spinal stenosis can prevent early recurrence of stenosis symptoms compared with decompression alone. This is beneficial for the patient. However, at the same time, we face some challenges, such as more blood loss, perioperative complications, and adjacent spondylosis. Ding et al [22] retrospectively analyzed 98 elderly patients with DLS who underwent intervertebral fusion. They found a perioperative complication rate of 34.7% in all patients. Of these patients, 11.2% had serious complications, and 31.6% had minor complications. Furthermore, in addition to problems related to medical comorbidities and surgical approach, another problem that affects surgical results is complications related to implants [24]. Most of these elderly patients suffer from osteoporosis, and implant failure is always a risk. In our study, six patients (25%) in the short-segment fusion group developed complications of varying degrees. Three patients developed incision infection. One patient developed venous thrombosis in the lower limbs due to long-term bed rest. This is undoubtedly very dangerous for elderly patients.

Ploumis et al [25] conducted a retrospective study on the imaging data of 78 patients with degenerative scoliosis. They found that hypertrophy of the ligamentum flavum, herniated disc, and overgrowth of bone were more likely to cause neural tube stenosis than scoliosis. Therefore, nonfusion decompression is considered a suitable treatment option for DLS with spinal stenosis [26]. Several studies have reported that conventional laminar opening decompression achieved similar outcomes as short-segment fusion. Cheng et al [27] stated that for patients with mild DLS, the outcomes were similar in the open decompression group and the fusion group. Masuda et al [13] reported that Japanese Orthopaedic Association scores improved from 5.9±1.6 to 10.0±2.8 and from 7.2±2.0 to 11.3±2.8 in the decompression and fusion groups, respectively. The difference between the two groups was not significant. It is worth noting, however, that open decompression surgery alone is highly invasive due to the disruption of the posterior spinal structures. This procedure may lead to medically induced spinal instability and persistent back pain [28]. Minimally invasive techniques have become an increasingly popular surgical procedure with unique advantages. PTED, as a minimally invasive procedure that preserves the posterior ligament complex and other biomechanical structures [29], may be a safe and effective treatment.

Sairyo et al [30] used finite element techniques to evaluate the biomechanical behavior of the endoscope after decompression. They found no negative impact on the mechanical stability of the lumbar spine. Compared with open decompression surgery, minimally invasive PTED has the advantages of local anesthesia, less soft tissue resection, and faster recovery [29]. It not only reduces perioperative complications but also avoids exacerbating existing instability by reducing the destruction of bone and soft tissue. Therefore, it may be a good option for elderly DLS patients with combined spinal stenosis. Madhavan et al [6] performed endoscopic foraminal decompression in 16 patients with 10° to 20° coronal plane deformities. The final prognostic scores of all patients improved significantly. Hasan et al [31] treated patients with lumbar stenosis with mild to moderate deformity using full-endoscopic and minimally invasive decompression. They found similar clinical outcomes in both groups during a 12-month follow-up period, and the endoscopic approach showed a lower complication rate. Jin et al [28] provided their experience with PTED for the treatment of elderly degenerative scoliosis with unilateral stenosis. They concluded that a satisfactory clinical outcome can be achieved with PTED in patients with a small Cobb angle and no severe rotation or lateral slip. In our study, the VAS and ODI scores of both groups were significantly improved at 1 year postoperatively compared with the preoperative scores. There was no statistically significant difference between the two groups. The difference was that the PTED group exhibited lower VAS back pain scores and ODI scores at one week postoperatively. This may result from the fact that PTED surgery causes less damage to the lumbar bone tissue and paravertebral muscles.

In addition, deformity correction is another important consideration. Short-segment fusion to a certain extent corrected the coronal imbalance and restored disc height. The segmental lordosis of the operated segments in the short-segment fusion group was also increased compared to the preoperative period. However, the increased segmental lordosis did not change the overall lumbar lordosis. The local deformity of patients in the short-segment fusion group was corrected to some extent compared to the PTED group. However, at one year postoperatively, there was no significant difference in symptoms between the two groups. In our study, a significant positive correlation between imaging findings and clinical outcomes was not found. We believe that in elderly patients presenting primarily with radicular pain, resolution of the painful irritation caused by stenosis should probably be the first consideration. In the end, a satisfactory excellent/good rating was achieved in both groups with no serious anesthetic complications. This also suggests the feasibility of PTED surgery. The surgical approach is individualized and should be decided by both the patient and the medical specialist. Therefore, the PTED procedure may be another safe and effective option for elderly patients when conventional surgery has high risks.

Our research also has certain limitations. First, the patient sample size was small, resulting in a limited ability to observe clinical results. Second, the learning curve of PTED surgery is high, and it is difficult for junior physicians to achieve adequate decompression of lumbar scoliosis. Finally, the observation time of this study was short, and long-term follow-up is needed.

## Conclusion

As the proportion of elderly people in the population increases, spine surgeons need to take into account the conditions of elderly patients, such as comorbidities, surgical risks, and health insurance costs. Both PTED and short-segment fusion have yielded good clinical outcomes in elderly DLS patients with Cobb angles of 10°-30° combined with spinal stenosis. However, PTED under local anesthesia was less invasive and had lower complication rates. Therefore, PTED may be an effective complement to conventional open surgery in elderly DLS patients with combined spinal stenosis.

## Data Availability

The datasets used and/or analysed during the current study are available from the corresponding author on reasonable request.

## Abbreviations

DLS: Degenerative lumbar scoliosis
PTED: Percutaneous transforaminal endoscopic discectomy
VAS: Visual analog scale
ODI: Oswestry disability index
